# Clinicopathological characteristics and prognosis of patients with IgA nephropathy and renal vasculitic lesions

**DOI:** 10.1186/s12882-021-02556-y

**Published:** 2021-10-28

**Authors:** Xueqing Tang, Qiong Wen, Qian Zhou, Wei Chen

**Affiliations:** 1 Department of Nephrology, The First Affiliated Hospital, Sun Yat-sen University, 58 Zhongshan Road II, Guangzhou, 510080 China; 2 Key Laboratory of National Health Commission, Guangzhou, 510080 China; 3 Guangdong Provincial Key Laboratory of Nephrology, Guangzhou, 510080 China; 4 Department of Nephrology, The First Affiliated Hospital of Shandong First Medical University & Shandong Provincial Qianfoshan Hospital, Jinan, 250014 China; 5 Clinical Trials Unit, The First Affiliated Hospital, Sun Yat-sen University, Guangzhou, 510080 China

**Keywords:** Clinicopathological characteristics, IgA nephropathy, Renal vasculitic lesions

## Abstract

**Background:**

We studied patients with IgA nephropathy (IgAN) and compared those with and without renal vasculitic lesions (RVLs).

**Methods:**

From January 2006 to December 2011, patients with biopsy-proven primary IgAN at our institution were retrospectively examined and assigned to an RVL group or a no-RVL group. RVLs were defined as thromboses in arteries and/or arterioles, necrosis of capillary loops, crescent formation, and fibrinoid necrosis of small blood vessels. The association of RVLs with clinical outcomes was analyzed using multivariate models. The primary composite endpoint was end-stage renal disease or doubling of serum creatinine.

**Results:**

There were 1570 patients, 50.2% (788) with RVLs and 49.8% (782) without RVLs. The RVL group was younger; had shorter disease course, more severe proteinuria and hematuria, worse renal function; and were prescribed more steroids and/or immunosuppressants. The RVL group had a greater prevalence of global glomerular sclerosis, more crescents, and a higher Oxford classification grade. A total of 501 patients in the RVL group (50.7%) and 487 in the no-RVL group (49.3%) completed follow-up. The RVL group was more likely to reach the composite endpoint after 1, 3, and 5 years (all *P* < 0.001). Proteinuria, anemia, low eGFR, and global and segmental sclerosis were independent predictors of progression to the composite endpoint in patients with RVLs.

**Conclusions:**

Almost half of our IgAN patients had RVLs, and these patients were younger and had worse renal function, with more severe proteinuria, hematuria, and severe pathologic lesions. IgAN patients with RVLs had worse renal outcomes than those without RVLs.

## Background

IgA nephropathy (IgAN) is the most common type of primary glomerulonephritis worldwide and accounts for about 45.3% of all cases of primary glomerulonephritis in Asia [1, 2]. Long-term follow-up studies demonstrated that the prognosis of patients with IgAN remains poor, in that 30 to 40% of them develop end-stage renal disease (ESRD) by 10 to 25 years after diagnosis [3]. In an effort to develop interventions that delay disease progression, many studies have examined the association of various risk factors with prognosis, including increased urinary protein level (> 1 g/day), decreased glomerular filtration rate (GFR), hypertension, hyperuricemia, male sex, and various clinical scores indicative of disease severity [4–6].

Renal histopathological lesions play an important role in guiding treatment decisions and predicting prognosis. The original 2009 Oxford Classification of IgAN indicated that higher scores for mesangial hypercellularity (M1 vs. M0), segmental glomerulosclerosis (S1 vs. S0), and tubular atrophy/interstitial fibrosis (T1/2 vs. T0) provided important prognostic information, in addition to the standard clinical features [7]. Other research reported that endocapillary hypercellularity (E1 vs. E0), was associated with the rate of renal functional decline, and was significantly reduced in patients receiving immunosuppressive therapy [8]. The 2016 Oxford Classification of IgAN recommended that a score for crescents (C1/2 vs. C0) also be considered for calculation of a MEST-C score [9]. However, the MEST-C score does not consider all the pathological changes that occur during IgAN. In particular, some of these patients may present with renal vasculitic lesions (RVLs).

A previous study reported that the prevalence of RVLs in patients with IgA nephropathy (including Henoch-Schönlein purpura and primary IgAN) was 18.5% (67/363), and that the 67 positive patients had a 5-year survival rate of 84% and a renal survival rate of 85%. Moreover, overall renal function, blood pressure at presentation, and chronic renal damage based on biopsy were major prognostic factors [10]. Glomerular crescents are a kind of RVL, and a large Korean cohort study of IgAN patients reported that the proportion of crescents in these patients had a linear association with adverse renal outcome, and that a C2 score of the MEST-C was a strong predictor of progression to ESRD and halving of the estimated GFR (eGFR) [11].

However, the prevalence of RVLs in patients with primary IgAN is still unclear. A large study is needed to identify the clinicopathological characteristics and other factors associated with clinical outcomes in IgAN patients who have RVLs. The present retrospective study analyzed the clinicopathologic features and outcomes of IgAN patients who presented with or without RVLs at a single center in China.

## Methods

From January 2006 to December 2011, 1570 patients (age ≥ 14 years) were diagnosed with primary IgAN at The First Affiliated Hospital, Sun Yat-sen University (Guangzhou, China). These diagnoses were based on mesangial IgA deposition from immunofluorescence microscopy as the major or one of the major immunoglobulins. Patients with Henoch-Schölein purpura, systemic lupus erythematosus, hepatitis B-related nephritis, and transplantation-related IgAN were excluded.

The demographic and clinical data of all patients, including age, gender, and use of medications, were collected at the time of the renal biopsy. Data on the composite renal outcome, defined as ESRD (eGFR < 15 mL/min/1.73 m^2^, dialysis, or renal transplantation) or doubling of the serum creatinine (SCr) level, were recorded during the follow-up. The additional laboratory data included urinary levels of protein and uric acid, blood levels of albumin and lipids, and other factors. The Modification of Diet in Renal Disease (MDRD) formula was used to calculate eGFR [12].

Kidney biopsy specimens were examined using light microscopy, immunofluorescence microscopy, and electron microscopy for assessment of the number of glomeruli and the presence of mesangial hypercellularity, glomerulosclerosis, glomerular crescents, endocapillary proliferation, tubular atrophy, and interstitial fibrosis. A biopsy was considered adequate if it contained 10 or more glomeruli. All specimens were also graded using the Oxford classification [8]. Here, we focus on patients with IgAN who also have RVLs. Based on renal histopathology, these lesions are characterized by thromboses in the arteries and/or arterioles, cell necrosis in the capillary loops, crescent formation, and fibrinoid necrosis of small blood vessels [10, 13, 14]. Each patient provided informed consent prior to the renal biopsy, and was assigned to the RVL group or the no-RVL group based on biopsy results.

### Statistical analysis

Quantitative parameters with normal distributions were expressed as means ± SDs and those with non-normal distributions as medians with interquartile ranges (IQRs). Parameters were compared using a one-way ANOVA or the Kruskal-Wallis test, as appropriate. The least significant difference (LSD) *t-*test, the Student-Newman-Keuls *q*-test, or the Bonferroni test was used for multiple comparisons, as appropriate. Differences in the qualitative results, expressed as frequencies with percentages, were compared using the chi-square test, Fisher’s exact test, or the Kruskal-Wallis test, as appropriate. The cumulative incidence of the reaching the composite endpoint was determined using the Kaplan-Meier method, and the log-rank test was used to compare the two groups. Factors significantly associated with the composite endpoint were identified using Cox regression analysis, and factors with *P*-values of 0.05 or less in the univariate analysis were entered into a multivariable Cox regression model. A backward elimination procedure (*P* > 0.10) was used to identify independent predictors of the composite endpoint. A difference was considered significant if the *P*-value was below 0.05. All statistical analyses were performed using SPSS software version 13.0 (SPSS Inc., Chicago, IL, USA, www.spss.com).

## Results

### Baseline clinicopathological features of IgAN patients with and without RVLs

We analyzed the baseline characteristics of 1570 patients with IgAN, 788 (50.2%) in the RVL group and 782 (49.8%) in the no-RVL group (Table 1). Overall, 598 patients (38.1%) had hypertension and 721 patients (45.9%) were male. The RVL group was significantly younger; had a shorter course of disease; had more severe proteinuria, hematuria, and hyperuricemia; and had lower levels of serum albumin, hemoglobin, and eGFR (all *p* < 0.05).

**Table 1:** Baseline clinicopathological features of all IgAN patients and of those with and without renal vasculitic lesions^a^

	All patients (*n* = 1570)	RVL Group (*n* = 788, 50.2%)	no-RVL Group (*n* = 782, 49.8%)	*P*-value^**^
Age (years)	32.0 (26.0,39.0)	31.0 (25.0,38.0)	32.0 (26.0,39.0)	**0.042**
Male	721,45.9%	379,48.1%	342,43.7%	0.083
Course of disease (months)	6.9 (2.1,24.8)	6.1(1.9,18.1)	8.7(2.3,25.9)	**0.002**
Hypertension	598,38.1%	300,38.1%	298,38.1%	0.988
Urinary protein (g/24 h)	0.6 (0.3,1.3)	0.7 (0.4,1.6)	0.5 (0.3,1.1)	**0.001**
Microscopic hematuria > 2+	264,16.8%	164,20.8%	100,12.8%	**< 0.001**
Serum albumin (g/L)	39.9 (36.0,42.2)	39.0 (35.0,42.0)	40.0 (37.0,43.0)	**0.009**
eGFR (mL/min/1.73 m^2^)	90.8 (54.5121.1)	85.4 (50.3114.3)	97.9 (61.0,126.5)	**< 0.001**
Hemoglobin (g/L)	127.0 (113.0,141.0)	125.0 (112.0,138.0)	128.0 (115.0,142.0)	**0.004**
Uric acid (μmol/L)	359.0 (274.5444.0)	370.0 (290.0,461.0)	344.5 (266.0,430.0)	**< 0.001**
LDL-C (mmol/L)	3.1 (2.5,3.8)	3.1 (2.5,3.9)	3.0 (2.4,3.8)	0.090
Serum IgA (g/L)	2.8 (2.3,3.5)	2.9 (2.3,3.5)	2.8 (2.3,3.5)	0.606
Renal biopsy results				
C0/C1/C2	901/605/64	119/605/64	782/0/0	**< 0.001**
Crescents (%)	0.0 (0.0, 7.1)	7.1 (3.6, 13.6)	0.0 (0.0, 0.0)	**< 0.001**
GS (%)	12.5 (2.55,34.8)	13.6(4.2,34.8)	10.7 (0.0,34.9)	**0.020**
M1	840,53.5%	461,58.5%	379,48.5%	**< 0.001**
S1	722,46.0%	421,53.4%	301,38.5%	**< 0.001**
E1	278,17.7%	189,24.0%	89,11.1%	**< 0.001**
T0/T1/T2	1080/390/100	512/223/53	568/167/47	**< 0.001**
RASI treatment	1175,74.8%	597,75.8%	578,73.9%	0.399
Steroid treatment	433,27.6%	283,35.9%	150,19.2%	**< 0.001**
Immunosuppressant^b^	28,1.8%	22,2.8%	6,0.8%	**0.003**

*RBC* Red blood cells, *HPF* High-power field (400×), *GS* Global glomerulosclerosis, *M1* Mesangial hypercellularity, *S1* Segmental glomerulosclerosis, *E1* Endocapillary hypercellularity, *RASI* Renin-angiotensin-system inhibitor, *eGFR* Estimated glomerular filtration rate, calculated as described by Ma et al. [12]

^**^P-values are from comparisons of the RVL and no-RVL groups

^a^Data are presented as N, % or median (IQR)

^b^Immunosuppressant included cyclophosphamide, mycophenolate mofetil and leflunomide

The RVL and no-RVL groups also had significant differences in Oxford classification scores. In particular, patients in the RVL group were more likely to have global sclerosis (GS), mesangial cell proliferation (M1 vs. M0), endothelial cell proliferation (E1 vs. E0), segmental glomerulosclerosis (S1 vs. S0), and tubular atrophy/interstitial fibrosis (T1/2 vs. T0) (all *p* < 0.05). More patients in the RVL group were also taking steroids and/or immunosuppressants.

After excluding patients who had baseline eGFR values below 15 mL/min/1.73 m^2^ or who were lost to follow-up, there were 988 patients (Table 2). The average follow-up time of these patients was 49 months, and there were 501 patients (50.7%) in the RVL group and 487 (49.3%) in the no-RVL group. These two groups had significant differences in all the same parameters as above (age, course of disease, etc.), except for M1 status, which was similar in the RVL and no-RVL groups (*P* = 0.811).

**Table 2:** Baseline clinicopathological features of all IgAN patients and of those with and without renal vasculitic lesions, after excluding patients who had low baseline eGFR (< 15 mL/min/1.73 m^2^) or were lost to follow-up^a^

	All patients (*n* = 988)	RVL Group (*n* = 501, 50.7%)	no-RVL Group (*n* = 487, 49.3%)	*P*-value^**^
Age (years)	32.0 (26.0,39.0)	31.0 (25.0,38.0)	32.0 (26.0,40.0)	**0.017**
Male	417,42.2%	225,44.9%	192,39.4%	0.081
Course of disease (months)	7.5 (2.2,24.8)	6.7(1.9,18.19)	9.8(2.5,28.9)	**0.001**
Hypertension	331,33.5%	167,33.3%	164,33.7%	0.909
Urinary protein (g/24 h)	0.6 (0.3,1.2)	0.7 (0.4,1.5)	0.5 (0.3,1.0)	**< 0.001**
Microscopic hematuria > 2+	655,66.4%	365,72.9%	291,59.8%	**< 0.001**
Serum albumin (g/L)	40.0 (36.4,42.7)	39.0 (35.9,42.0)	40.0 (37.0,43.0)	**0.002**
eGFR (mL/min/1.73 m^2^)	96.1 (61.6123.7)	88.7 (57.9117.1)	105.7 (69.4128.9)	**< 0.001**
Hemoglobin (g/L)	127.0 (115.0,140.0)	125.0 (113.0,138.0)	128.0 (116.0,141.0)	**0.014**
Uric acid (μmol/L)	344.0 (268.0,429.0)	363.0 (282.0,450.0)	324.0 (255.3414.8)	**< 0.001**
LDL-C (mmol/L)	3.0 (2.5,3.7)	3.1 (2.5,3.9)	2.9 (2.4,3.6)	0.061
Serum IgA (g/L)	2.8 (2.3,3.5)	2.9 (2.3,3.6)	2.8 (2.3,3.5)	0.297
Renal biopsy results				
C0/C1/C2	562/387/39	75/387/39	487/0/0	**< 0.001**
Crescents (%)	0.0 (0.0, 7.1)	7.1 (3.7, 13.5)	0.0 (0.0, 0.0)	**< 0.001**
GS (%)	10.5 (0, 29.2)	11.9(3.6, 30.0)	8.7 (0.0, 27.8)	**0.015**
M1	949,96.1%	480,95.8%	469,96.3%	0.811
S1	623,63.1%	327,65.3%	296,60.8%	**< 0.001**
E1	224,22.7%	127,25.3%	97,19.9%	**0.004**
T0/T1/T2	506/478/4	233/266/2	273/212/2	**0.003**
RASI treatment	561,56.8%	280,55.9%	281,57.7%	0.571
Steroid treatment	503,50.9%	272,54.3%	231,48.0%	**< 0.001**
Immunosuppressant	26,2.6%	22,4.4%	4,0.8%	**0.003**

^**^P-values are from comparisons of the RVL and no-RVL groups

^a^Data are presented as N (%) or median (IQR)

### Outcome of IgAN patients with and without RVLs

**Fig. 1:**
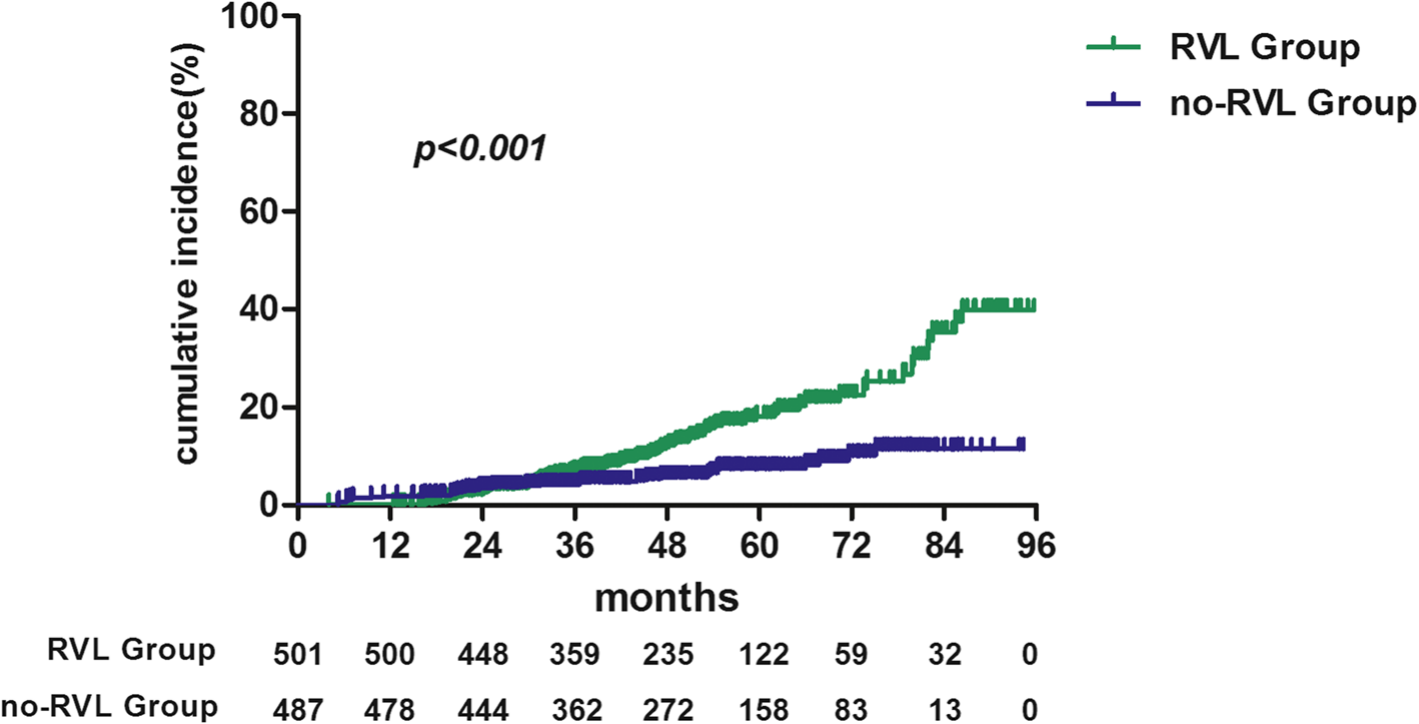
Kaplan-Meier analysis of the cumulative incidence of the composite endpoint in patients with renal vascular lesions (green, top) and without renal vascular lesions (blue, bottom)

During the follow-up (mean: 49 months; interquartile range [IQR]: 35 − 63 months), the 1-year, 3-year, and 5-year incidence of the composite renal endpoint were 0.2, 6.8, 18.1% in the RVL group and 1.8, 4.2, 7.5% in the no-RVL group (Fig. 1). These differences were statistically significant (all *P* < 0.001).

We then analyzed the data using Cox regression analysis (Table 3). An unadjusted analysis and an analysis that adjusted for age, gender, and course of disease (Model 1) showed that the presence of RVLs was associated with the composite endpoint (both *P* < 0.001; Table 3). However, adjustment for all Model 1 factors as well as HBP, albumin, urinary protein, hemoglobin, LDL-cholesterol, uric acid, eGFR, M1, E1, S1, and T1/2 (Model 2) indicated this relationship was no longer significant (*P* = 0.07).

**Table 3:** Cox regression analysis (unadjusted, Model 1^a^, and Model 2^b^) of the relationship of renal vasculitic lesions with the composite renal outcome

	Unadjusted		Model 1		Model 2	
	HR (95%CI)	*P*-value	HR (95%CI)	*P*-value	HR (95%CI)	*P*-value
No-RVL Group	Ref.		Ref.		Ref.	
RVL Group	2.31 (1.53, 3.49)	**< 0.001**	2.30 (1.52, 3.49)	**< 0.001**	2.15 (0.93, 4.99)	0.07

^a^Model 1: adjusted for age, gender, and course of disease

^b^Model 2: adjusted for age, gender, course of disease, HBP, albumin, urinary protein, hemoglobin, LDL-cholesterol, uric acid, eGFR, M1, E1, S1, and T1

### Factors associated with outcome in IgAN patients with RVLs

We also analyzed the association of multiple factors with the composite endpoint in the 501 IgAN patients who had RVLs (Table 4). The Cox multivariate regression analysis found that progression to the composite endpoint was positively associated with proteinuria (hazard ratio [HR] = 1.256; 95% CI, 1.009–1.563; *P* = 0.041), GS (HR = 1.023; 95% CI = 1.010–1.036; *p* = 0.001), and S1 (HR = 2.315; 95% CI = 1.301 − 4.118; *p* = 0.004), and negatively associated with hemoglobin (HR = 0.982; 95% CI = 0.969–0.997; *P* = 0.015) and eGFR (HR = 0.977; 95% CI = 0.964–0.991; *P* = 0.001).

**Table 4:** Multivariate analysis of factors associated with the composite renal endpoint in IgAN patients with renal vasculitic lesions

Variable	HR	95% CI	*P*-value
Age (years)	0.979	0.948–1.011	0.191
Female	1.415	0.776–2.581	0.258
Course of disease (months)	0.993	0.983–1.004	0.221
Hypertension	0.755	0.393–1.447	0.397
Urinary protein (g/24 h)	1.256	1.009–1.563	0.041
Serum albumin (g/L)	0.997	0.935–1.062	0.915
Hemoglobin (g/L)	0.982	0.969–0.997	0.015
Uric acid (μmol/L)	1.000	0.997–1.003	0.960
LDL-C (mmol/L)	1.145	0.932–1.406	0.196
eGFR (mL/min/1.73 m^2^)	0.977	0.964–0.991	0.001
Global sclerosis (%)	1.023	1.010–1.036	0.001
M1	1.338	0.771–2.324	0.301
E1	1.213	0.655–2.249	0.539
S1	2.315	1.301–4.118	0.004
T1-2	1.010	0.623–1.638	0.968

## Discussion

In this study, more than half of the IgAN patients who presented to our hospital over a 6 year period had RVLs, more than reported by Pankhurst et al. [10], who reported this condition in only 67 of 363 (18.5%) IgAN patients. Another retrospective cohort study from China found that 20.6% (194/944) of adult IgAN patients had renal arteriolar microangiopathic lesions (including endothelial cell swelling, subintimal edema, arteriolar thrombosis, fibrinoid necrosis, and arterial fibrous intimal thickening with concentric lamination) [15]. In contrast, Khalil et al. [16] indicated that 53% (68/128) of IgAN patients presented with lesions characterized as thrombotic microangiopathy (TMA), acute or organized, in renal arteries, and/or arterioles. The discrepancies among these studies might be due to different sample sizes, different study populations, and different definitions of RVL. For example, Pankhurst et al. [10] did not include patients with segmental thrombosis, disruption of glomerular capillary loops, and crescent formation in the glomerulus. The definition of microangiopathic lesions in the study of Cai et al. [15] emphasized endothelial, intimal, and subintimal lesions in the renal arterioles, and did not include cell necrosis in the capillary loops or crescent formation. However, our study paid more attention to pathologic changes of glomerular vasculitis.

Furthermore, our comparison of patients with and without RVLs indicated that the RVL group was younger, had greater proteinuria and hematuresis, and had a higher uric acid level, but had lower levels of serum albumin, hemoglobin, and eGFR. Our RVL group also had more GS and more severe damage from mesangial cell proliferation, endothelial cell proliferation, segmental glomerulosclerosis, and tubular atrophy/interstitial fibrosis. The previous Chinese cohort study of Cai et al. [15] showed that patients with microangiopathic lesions had higher blood pressure, more severe proteinuria, higher serum uric acid level, and lower eGFR. These researchers also found that the group with microangiopathic lesions had a greater Oxford pathologic score and a greater proportion of patients with GS [15]. Khalil et al. [16] also reported that their group with TMA had greater proteinuria, lower serum albumin, higher SCr, lower eGFR at the time of the biopsy, a greater percentage of sclerotic glomeruli, more severe tubulointerstitial fibrosis, and a greater prevalence of hypertension (71.0% vs. 23.3%) [16]. In contrast, our RVL and no-RVL groups had similar prevalences of hypertension. This difference may be because these two previous studies focused on microangiopathic lesions, rather than renal vasculitic lesions in general.

In addition, most of the patients in our RVL group received treatment with steroids and/or immunosuppressants. This result is consistent with Cai et al. [15], who also showed that more patients with microangiopathic lesions received steroids and/or other immunosuppressants (45.9% vs. 33.3%; *P* = 0.001) [15]. However, we did not examine whether this treatment preserved renal function and prevented the progression to ESRD. Harper et al. showed that treatment of patients with IgAN with steroids and/or immunosuppressants led to healing of the vasculitic lesions and thus may have prevented the progression of glomerular scarring [13].

Patients with IgAN typically have poor prognoses, in that 25 to 30% of them develop progressive renal failure at 20 to 25 years after the initial diagnosis, and an estimated that 1 to 2% of adults develop ESRD each year [17, 18]. A small number of previous studies examined the outcomes of patients with vasculitic IgAN. For example, Pankhurst et al. reported that after 5 years, the patient survival rate was 84% and the renal survival rate was 85% [10]. Similarly, the 5-year renal survival rate was 81.9% in our RVL group. However, Cai et al. reported that after a median follow-up time of 4.2 years, 75 (38.7%) patients with microangiopathic lesions reached the composite renal endpoint [15]. These results may be discrepant in part because Cai et al. used a composite renal end point that included a 50% reduction in eGFR or death, not simply ESRD. Because very few of our patients died and we do not have long-term follow-up data, we did not consider patient survival as a study endpoint. Moreover, although we found that RVL was a risk factor for reaching the composite renal endpoint, our Cox regression analysis indicated that RVL was not a significant and independent risk factor. In contrast, Cai et al. showed that the presence of microangiopathic lesions was an independent risk factor for kidney failure after adjusting for clinical and pathologic variables measured at biopsy (HR = 1.95; 95% CI = 1.34–2.83) [15].

The risk factors associated with renal outcome in IgAN patients with RVLs remain incompletely understood. Our results indicated that proteinuria, low hemoglobin, low eGFR, global sclerosis, and S1 were independently associated with progression to the composite renal endpoint. Pankhurst et al. similarly reported that poor renal function, high blood pressure at presentation, and greater chronic renal damage based on biopsy results were associated with poor renal outcome [10]. However, proteinuria at presentation was unrelated to outcome in this previous study. Notably, most (52/67) vasculitic patients in the Pankhurst et al. study [15] received treatment with a steroid and/or immunosuppressant, and this may be why they found no association of proteinuria with renal outcome.

This study has several limitations. First, this was a retrospective study and the patients were from a single university hospital in China. Thus, validation of our results in different populations or by use of a prospective study is necessary. Second, many of our patients were lost to follow-up (more than 30%). Third, we did not assess death as a hard endpoint because very few patients died (9 in the RVL group and 4 in the no-RVL group) due to our relatively short follow-up time. Thus, we could not perform a complete assessment of the clinical outcomes in our two groups. Finally, we are unable to assess the impact of treatment on outcome, because we did not analyze data on treatment details. Therefore, a multi-center study with a long-term follow-up, and prospective cohort design should be performed.

## Conclusions

In conclusion, IgAN patients with RVLs were younger and had more severe proteinuria and hematuria, worse renal function, more severe pathologic lesions, and worse renal outcome. Proteinuria, anemia, lower eGFR, and global and segmental sclerosis were independent predictors of poor renal outcome in IgAN patients with RVLs.

## Data Availability

The datasets generated and analyzed during the current study are not publicly available due to none of the data types requiring uploading to a public repository but are available from the corresponding author on reasonable request.

## Abbreviations

IgAN: IgA nephropathy
ESRD: End-stage renal disease
GFR: Glomerular filtration rate
RVLs: Renal vasculitic lesions
eGFR: Estimated GRF
IQRs: Interquartile ranges
LSD: Least significant difference
